# An Inexpensive and Accurate Reverse Transcription-PCR–Melting Temperature Analysis Assay for Real-Time Influenza Virus B Lineage Discrimination

**DOI:** 10.1128/JCM.00602-19

**Published:** 2019-11-22

**Authors:** Fernando Couto Motta, Priscila Silva Born, Paola Cristina Resende, David Brown, Marilda Mendonça Siqueira

**Affiliations:** a WHO/NIC, Respiratory Viruses and Measles Laboratory, Oswaldo Cruz Institute, Fiocruz, Rio de Janeiro, Brazil; Memorial Sloan Kettering Cancer Center

**Keywords:** influenza B, Yamagata lineage, Victoria lineage, melting temperature, melting curve, RT-PCR

## Abstract

In this work, we describe a SYBR-Green one-step reverse transcription-PCR protocol coupled with a melting temperature analysis (RT-PCR-*T_m_*), which allows the discrimination of influenza B lineages Yamagata and Victoria. The assay is performed using a regular real-time thermocycler and is based on differences in melting temperature (*T_m_*) of a 131-bp amplicon, obtained from a conserved region of hemagglutinin gene. A total of 410 samples collected during the 2004, 2008, and 2010–2017 influenza seasons in Brazil were tested, and the lineages were correctly characterized using their melting profiles. The temperature range is significantly different between both lineages throughout the time (Mann-Whitney test; *P* < 0.0001, confidence interval = 95%), and the *T_m_* is not affected by viral load (Spearman correlation test; *r* = 0.287, *P* = 2.245 × 10^−9^). The simplicity and cost-effectiveness of this protocol make it an option for influenza B lineage surveillance worldwide.

## Abstract (precis)

In this work, we describe a SYBR-Green one-step reverse transcription-PCR protocol coupled with a melting temperature analysis (RT-PCR-*T_m_*), which allows the discrimination of influenza B lineages Yamagata and Victoria. The assay is performed using a regular real-time thermocycler and is based on differences in melting temperature (*T_m_*) of a 131-bp amplicon, obtained from a conserved region of hemagglutinin gene.

## INTRODUCTION

Influenza is a contagious respiratory disease which accounts for 300,000 to 650,000 severe cases annually, causing important burden to national health services worldwide (1). This disease has a higher prevalence during the winter season in temperate regions and a less marked but more complex distribution in tropical and equatorial areas (2, 3). The etiologic agents are the influenza viruses, a group of segmented single-stranded RNA viruses classified in the *Orthomyxoviridae* family and divided in Influenza types A, B, and C and the recently described type D (4). Influenza types A and B are major causes of impact on public health. Influenza B is responsible for fewer cases of disease in most seasons, but a significant proportion of cases are attributed to this type, annually (5), especially between children and teenagers, at the end of the season (6, 7).

Two distinct influenza B lineages have been cocirculating since their identification in the late 1980s (8), being known as the Victoria (Vic) and Yamagata (Yam) lineages, named after their prototypes strains B/Victoria/2/87 and B/Yamagata/16/88 (9). Although genetic and antigenically more stable than influenza A, the influenza B viruses have great diversity, since distinct strains in both lineages can cocirculate for longer periods of time in comparison to influenza A (6). The Yam and Vic lineages are both genetically and antigenically distinct. A molecular protocol for lineage accurate differentiation, suitable for use in epidemic seasons, is mandatory for an up-to-date epidemiologic service and ultimately important to support World Health Organization (WHO) vaccine recommendations.

After the 2009 pandemic, influenza surveillance networks in Brazil adopted a fluorescent-probe hydrolysis reverse transcription-PCR (RT-PCR) assay, developed by the WHO Influenza Collaborating Centre for the Surveillance, Epidemiology, and Control of Influenza at Centers for Disease Control and Prevention (WHOCC-CDC; Atlanta, GA) for influenza molecular diagnostic. This is a standard TaqMan assay used for real-time detection and characterization of Influenza A and its currently human circulating subtypes (H1N1pdm09 and H3N2) and influenza B (10). Despite the progress on molecular subtyping of influenza A, the characterization of influenza B lineages remained for long time based on the labor-intense HA inhibition assay performed with isolated samples or on phylogenetic analysis of HA gene sequences (11). Recently, real-time RT-PCR protocols to differentiate influenza B lineages directly from clinical specimens have been made available, improving the throughput of the process. However, these protocols are based on expensive MGB probes (12) or proprietary report molecules (13), requiring pyrosequencing capacity (14), or need a multiple-step assay (15).

Previously, we presented a denaturing gradient gel electrophoresis (DGGE) protocol suitable to differentiate the influenza B lineages based on the melting temperature (*T_m_*) of a specific fragment of the HA region (16). This previous protocol is accurate and reproducible but also laborious and dependent on DGGE capability. In the present study, we describe a straightforward, economical, and fast one-step RT-PCR protocol that allows the specific detection and differentiation of Yam and Vic influenza B lineages, based on the different melting characteristics of a 131-bp amplicon from a conserved region of the HA gene. Our results present an influenza B lineage discrimination protocol based on melting curve analysis, as an alternative to probe-hydrolysis protocols available until now.

## MATERIALS AND METHODS

### Virus isolates, clinical specimens, RNA extraction, and ethics.

Two influenza B strains isolated in Madin-Darby canine kidney (MDCK) cells, Yam (B/Bahia/368/2011) and Vic (B/Sergipe/435/2011), were used as lineage prototypes. Infectious viruses in culture supernatants were quantified by determining the number of 50% tissue culture infectious doses per ml (TCID_50_/ml), and 10-fold serial dilutions of these viruses were used for assay standardization. In order to verify the limit of detection (LoD) of the assay, serial dilutions of a modified synthetic RNA (RNA Ultramer; IDT) were used. A total of 410 clinical influenza B-positive specimens, collected at Brazilian State Public Health Laboratories during 2004, 2008, and 2010 to 2017 and characterized in our laboratory (National Influenza Centre, FIOCRUZ, Rio de Janeiro, Brazil) were used in order to check the SYBR RT-PCR protocol suitability for lineage differentiation. The samples had different origins: 355 nasopharyngeal aspirates or nasopharyngeal-throat combined swabs were from patients with influenza-like illness, 47 nasopharyngeal aspirates and 1 lower respiratory tract aspirate were from in-patients with severe respiratory infection, and 7 clinical strains were isolated in MDCK cells. All samples were also characterized as Vic or Yam lineages using the WHOCC-CDC real-time RT-PCR protocol for lineage differentiation (www.cdc.gov/flu/clsis). In addition to the real-time protocols, 174 samples were further characterized as Yam (*n* = 72) or Vic (*n* = 102) by Sanger sequencing of the HA gene. The sequences are available from Global Initiative on Sharing All Influenza Database (GISAID; https://www.gisaid.org). The viral RNA of clinical and isolated samples was extracted using QIAmp RNA viral minikit (Qiagen, Hilden, Germany) according to the manufacturer’s protocol. The RNA was eluted in 80 μl and conserved at –80°C until use. This study was approved by the FIOCRUZ-IOC Ethics Committee (68118417.6.0000.5248) and the Brazilian Ministry of Health SISGEN (A1767C3).

### Primers design and amplicon conservation.

Influenza B-specific primers were designed targeting the end of HA1 domain on the HA gene, a region that exhibits a distinct *T_m_* for each lineage, as previously described (16). Primers BHA739f (5′-CCA RAT CAA ACA GAA GAC GGA G-3′) and BHA869r (5′-CAC CAC ACY TTT TGA GGC AA-3′) generated a 131-bp amplicon for both lineages. The number in the primer designations refers to the B/Victoria/2/87 HA gene numbering with a signal peptide. The specificity of these primers was verified using the Nucleotide Basic Local Alignment Search Tool (BLAST; http://blast.ncbi.nlm.nih.gov/Blast.cgi) in order to avert nonspecific reactivity. The conservation of primers’ targeted regions, as well as the key residues for *T_m_* differentiation between lineages over time, was checked with sequences for each lineage available using the GISAID platform. The sequences were aligned and analyzed using Geneious 7.1 software, and the frequency of each nucleotide by residue throughout Yam and Vic alignments was determined using a Python script (fasta_bases_counter; https://github.com/LVRS).

### Melting calculations.

Sequences produced in our laboratory and other available in GISAID database were used to evaluate the melting domains and their conservation inside and between lineages. The theoretical overall helicity (likeliness to remain a double strain) of the amplicon as a function of temperature for low-, average-, and high-*T_m_* representatives inside each lineage was verified using the on-line application U-Melt (17). The thermal stability and the denaturation behavior of melting domains inside the 131-bp amplicon were graphically assessed using an algorithm (18) implemented in the web-based application Poland (http://www.biophys.uni-duesseldorf.de/local/POLAND/poland.html).

### Real-time SYBR green RT-PCR protocol and melting temperature analysis.

Real-time SYBR RT-PCR, followed by melting curve analysis, was performed using an ABI StepOnePlus thermocycler. The reaction was performed using a QuantiFast SYBR RT-PCR kit (Qiagen) according to the manufacturer’s instructions, with a total reaction volume of 20 μl (15 μl of reaction mix plus 5 μl of template RNA) and the specific designed primers. The RNA obtained directly from clinical samples was tested in triplicates, along with the positive-control isolates B/Bahia/368/2011 and B/Sergipe/435/201, which were deployed in dilutions from 10^−1^ to 10^−3^ in every reaction plate. The cycling conditions were as follows: 50°C/10 min (reverse transcription) and 95°C/5 min (polymerase activation), followed by 40 cycles at 95°C/10 s and 57°C/30 s, with data acquisition at 57°C anneal/extension step. The dissociation curve stage was performed immediately after the last PCR cycle, at 95°C/15 s (preliminary denaturation) and an initial hold step of 70°C/1 min, followed by a 0.3°C increment up to 85°C. The total cycling time is around 80 min.

## RESULTS

**FIG 1:**
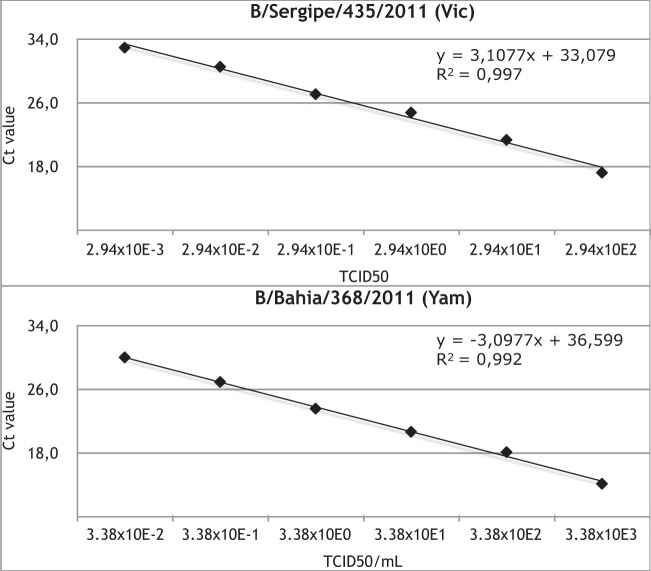
Reaction efficiencies of the SYBR green RT-PCR for influenza B detection. The SYBR green RT-PCR assay was evaluated by testing 6-fold serial dilutions (triplicate reactions) of RNA from B/Sergipe/435/2011 (Vic lineage control) and B/Bahia/368/2011 (Yam lineage) isolated virus stocks. Mean *C_T_* values were plotted against the TCID_50_ dilutions. The slope and *R*^2^ values for each lineage are shown in the graphs.

The sensitivity of the protocol was evaluated using triplicates of 10-fold serial dilutions of total RNA retrieved from B/Bahia/368/2011 (Yam) and B/Sergipe/435/2011 (Vic) isolated virus stocks, with 33.804 TCID_50_/ml (HA = 1:256/25.0 μl, 0.5% turkey red cells) and 2.944 TCID_50_/ml (HA = 1:32/25.0 μl, 0.5% turkey red cells), respectively. The assay still presented clear positive results for both lineages up to 10^−6^ dilution (Fig. 1). At this dilution, the threshold cycle (*C_T_*) values were 29.9 (3.38 × 10^−2^ TCID_50_) for Yam and 33.0 (2.94 × 10^−3^ TCID_50_) for Vic positive-control strains. The estimated amplification rates were 2.09 (R^2^ = 0.997) and 2.10 (R^2^ = 0.992) for Yam and Vic, respectively. The intra-assay standard deviations (SD) were 0.06 (mean *C_T_* = 20.8) for Yam and 0.09 (24.0) for Vic positive-control strains (see Fig. S1A in the supplemental material). The variability between the different runs was assessed using *C_T_* values of 10^−1^ to 10^−3^ dilutions of both controls. The *C_T_* values of intra- and interassays for both controls and their dilutions are summarized in the Table 1. A quantified synthetic RNA (RNA Ultramer; IDT) was used to verify the LoD of the protocol. The minimum RNA copy number accurately detectable was 60 copies per 20-μl reaction (Table 2). The absence of cross-reactivity was confirmed by analyzing real-time RT-PCR- and PCR-positive samples for some common human respiratory viruses. We found no positivity for the seasonal influenza A subtypes (H1N1)pdm09 and (H3N2); adenovirus type 2; respiratory syncytial viruses A and B; parainfluenza viruses 1, 2, and 3; human rhinovirus; enteroviruses 6 and 71; or human metapneumovirus (data not shown).

**TABLE 1:** Evaluation of intra-assay and interassay variability

Comparison^*a*^	SD (avg *C_T_*)^*b*^					
	B/Sergipe/435/2011 (Vic)			B/Bahia/368/2011 (Yam)		
	10^−1^	10^−2^	10^−3^	10^−1^	10^−2^	10^−3^
Intra-assay	ND	ND	0.09 (24.0)	ND	ND	0.06 (20.8)
Interassay	1.42 (17.3)	1.43 (21.5)	0.60 (25.0)	0.58 (14.8)	0.52 (19.1)	0.27 (22.4)

a Intra-assay *C_T_* values represent the means of 39 replications; interassay *C_T_* values represent the means of five different runs performed over 6 months.

b SD and average *C_T_* values (in parentheses) are shown for Vic and Yam lineage control samples in serial dilutions. The original values (in TCID_50_/ml) were as follows: Yam, 3.38 × 10^3^; and Vic, 2.94 × 10^2^. ND, not determined.

**TABLE 2:** LoD assay findings determined using quantified RNA^*a*^

Copy no.	Mean *C_T_*
6,000	28.89
600	33.76
60	37.18
6	ND

a *C_T_* values represent the means of two different runs performed in duplicate. The quantified synthetic modified RNA fragment (RNA Ultramer; IDT) resembles the amplicon generated using this protocol.

### Comparison between CDC RT-PCR and RT-PCR-*T_m_* for influenza B detection.

We compared the SYBR green results to those obtained using the Centers for Disease Control and Prevention (CDC) standard protocol for influenza B detection. All 410 samples were tested in both assays simultaneously in order to minimize variations. The mean *C_T_* values obtained for influenza B-positive samples were 27.24 (SD = 5.08; range, 10.74 to 38.42) and 27.92 (SD = 4.58; range, 12.93 to 38.46) for our SYBR-Melt and the CDC probe-hydrolysis assays, respectively. The amplification curves of our SYBR green based protocol were well shaped (Fig. S1A), and the average *C_T_* values for Vic-like samples were slightly lower than those obtained for Yam-like ones in both protocols. The dispersion graph generated by plotting the *C_T_* differences between the CDC protocol and the proposed protocol against the average of both methods (19) indicates 94.4% agreement between the CDC RT-PCR protocol and our RT-PCR-*T_m_* protocol, with 387 of 410 samples inside the recommended limit range (see Fig. S2 in the supplemental material). Sanger sequences of the HA genes from 174 samples were used to verify both the CDC and the proposed *T_m_* protocols. Phylogenetic classification of the Vic and Yam lineages matched perfectly the real-time results. The list of samples and their respective nucleotide sequences (GISAID IDs) can be found in Table S1 in the supplemental material.

### *T_m_* discrimination of Victoria and Yamagata lineages.

**FIG 2:**
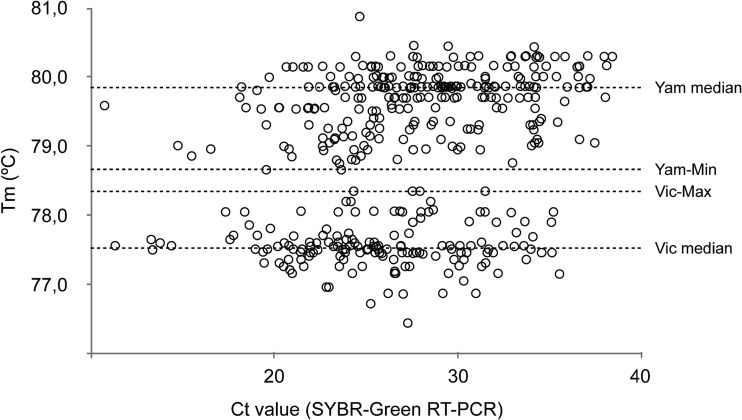
Dispersion of melting temperature values as a function of *C_T_* values in an RT-PCR-*T_m_* assay. The melting temperatures (*T_m_*s) of 410 samples tested with the RT-PCR-*T_m_* protocol as function their *C_T_* values. Samples were distinguished as Yamagata or Victoria lineage based on their melting behavior. The median *T_m_*s for each group, Yamagata minimum (78.7°C) and Victoria maximum (78.3°C), are indicated. The difference between the lowest Yamagata and the highest Victoria *T_m_* values was 0.3°C.

After the PCR stage, a melting curve was developed to differentiate the lineages based on their *T_m_* profiles. A single peak was detected for each sample during the melting curve analysis throughout all assays for both lineages. The average *T_m_* values obtained from the standard isolates B/Sergipe/435/2011 (Vic) and B/Bahia/368/11 (Yam) were 77.6 and 79.0°C, respectively (see Fig. S1B in the supplemental material). Of the total samples, 256 were characterized as Yam-like and 154 were characterized as Vic-like using the CDC RT-PCR protocol and sequencing. Using the SYBR-Melt protocol, Yamagata-like samples presented a *T_m_* ranging from 78.7 to 80.9°C (mean, 79.7°C; SD, 0.43) in melting-curve analysis, and the Victoria-like viruses ranged between 76.4 and 78.3°C (mean, 77.5°C; SD, 0.33), indicating an average distance of 1.2°C between lineages (Fig. 2). The smallest distance (∼0.3°C) was found between four Vic and two Yam samples collected in 2017. The temperature difference between both groups was statistically significant (Mann-Whitney test, *P* < 0000.1) and the *T_m_* was not affected by the *C_T_* of the sample (Vic, −0.1771; Yam, 0.1153 [Pearson’s correlation]).

### Evaluation of key residues for Victoria and Yamagata *T_m_* differentiation.

**FIG 3:**
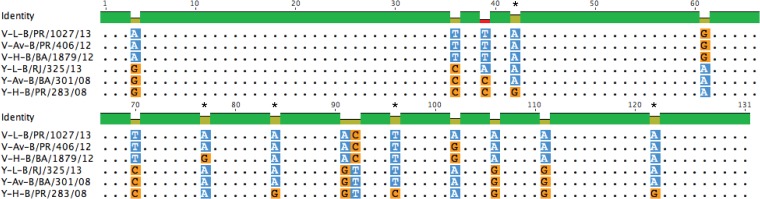
Placement of the nucleotide residues involved in Yamagata and Victoria low-, average-, or high-*T_m_* profiles. The alignment shows the placement of 15 nucleotide residues involved in *T_m_* differentiation in the chosen amplicon. Ten residues (4, 36, 39, 61, 70, 91, 92, 102, 106, and 111) allowed *T_m_* differentiation between the Victoria and Yamagata lineages. The other five residues (*) are associated with minor alterations in *T_m_*, generating the low- or high-*T_m_* profiles inside the lineage groups. Blue squares, weak interactions; orange squares, strong interactions. V–, Victoria; Y–, Yamagata; L–, low *T_m_* sequence; Av–, average *T_m_* sequence; H–, high *T_m_* sequence. The color bar over the alignment indicates conserved nucleotides, depicted as dots. The corresponding GISAID accession numbers are B/PR/1027/13-331397, B/PR/406/12-331414, B/BA/1879/12-331415, B/RJ/325/13-331462, B/BA/301/08-75226, and B/PR/283/08-75250.

**FIG 4:**
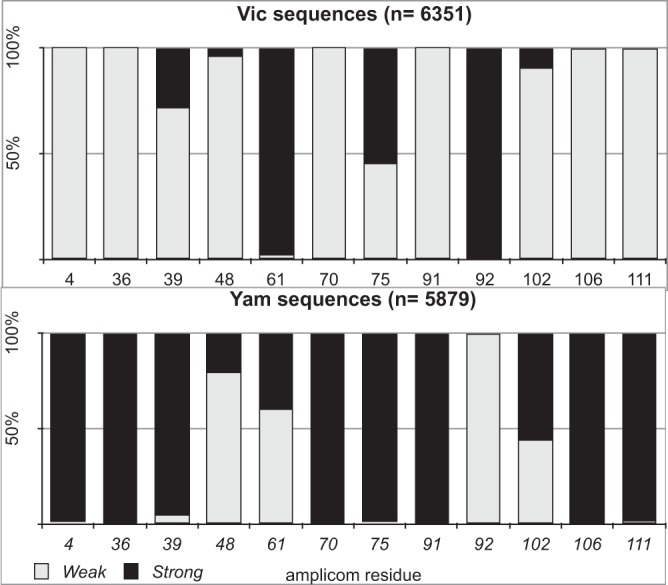
Frequencies of strong and weak interactions in key residues in the 131-bp amplicon used for influenza B Victoria and Yamagata lineage discrimination. Bars represent the percentage of weak (gray) or strong (black) interactions observed in the SYBR green RT-PCR 131-bp amplicon along all influenza B sequences available at GISAID from 2008 to 2018. Residues 4, 36, 70, 75, 91, 106, and 111 (amplicon count) are always strong at Yamagata-like samples. Residues 39, 48, 61, 75, and 102 are mixed, producing *T_m_* variances inside lineages. Only residues 61 and 92 contribute to the increment Victoria sequences *T_m_*. Except for the Victoria or Yamagata strains with A48G and the Victoria strain bearing A75G changes, found in the general alignment, all other combinations were observed in the samples analyzed in this study (Fig. 3). The graphs were developed using 5,879 Yamagata and 6,351 Victoria HA sequences (totaling 12,230 sequences). The base accounts/percentages by each residue were produced using a Python script (“fasta_bases_counter”) available at https://github.com/LVRS.

In order to identify, in the amplicon used, the nucleotide residues associated with *T_m_* differences inside and between Vic and Yam, we compared the differences between sequences presenting low, average, and high *T_m_* values from both lineages (Fig. 3). Of 15 different residues found between Vic and Yam, 10 were consistently different between them: 4, 36, 39, 61, 70, 91, 92, 102, 106, and 111. The other five differences are likely associated with the *T_m_* range observed in samples from the same lineage. Investigating the role of individual residues in the thermal stability of the amplicon, we identified a 30-base region in the middle that showed similar *T_m_* values for both lineages; this was likely due to residues 61 and 70, which neutralize each other. The differences between lineages and “low-*T_m_*” and “high-*T_m_*” variants are mainly concentrated at the 3′ extremity of the amplicon (see Fig. S3 in the supplemental material). Comparing sequences with a distinct *T_m_* inside lineages, we found a range between two (Vic-low/PR/1027/2013) to four (Vic-high/BA/1879/2012) and six (Yam-low/RJ/325/2013) to eleven (Yam-high/PR/283/2008) strong interactions (G-C) for Vic and Yam lineages, respectively (Fig. 3). This difference explains the higher stability, as well as the wider *T_m_* range, of Yam-like amplicons (see Fig. S4 in the supplemental material). Finally, we aligned all Yam and Vic HA sequences collected throughout the 2008–2017 period available on GISAID, and then we used a Python script to check the prevalence of each residue throughout the time. Twelve of the fifteen identified residues that were variable in the great alignment were classified as weak (A or T) or strong (C or G) interactions (Fig. 4). All *T_m_* residues involved in the distinction between influenza B lineages were found well conserved among the 6,350 Victoria and 5,879 Yamagata HA sequences evaluated.

## DISCUSSION

Influenza B lineage classification is based on the HA gene, the most variable segment of the virus genome. The HA nucleotide difference between Vic and Yam lineages is around 11% for recent samples. However, such differences are scattered throughout the HA gene, making it difficult to find annealing regions to differentiate Vic and Yam lineages, hampering the development of probe hydrolysis protocols. The method we presented here overcomes this constriction by using the *T_m_* of the amplicon, instead of its sequence, to distinguish the Yam and Vic lineages. In addition, since the assay is based on SYBR green chemistry, it is simple and inexpensive to perform, avoiding the need for probes for detection. Two protocols using a similar approach for influenza B lineage characterization have been published (15, 20). In comparison to these assays, the protocol described here presents a broader *T_m_* distance between Vic and Yam samples (especially comparing the high Vic *T_m_* with the low Yam *T_m_*), with less handling time (i.e., a one-step RT-PCR protocol) and simpler analysis. It still presents reliable melting curves without spurious peaks, even for samples with *C_T_* values around 35 in the CDC protocol. Importantly, this assay was not affected by the HA 162-163 or 162-164 deletions reported for some Victoria samples from the 2017–2018 period, since the amplicon here remains unchanged in a conserved area of the gene. In addition, the results obtained with this SYBR green assay are comparable to those achieved using the standard probe hydrolysis CDC method, presenting a similar ability to detect influenza B in clinical samples. Since 2017, this protocol has been integrated into our routine for influenza B lineage characterization, yielding results similar to phylogenetic analysis and CDC RT-PCR for samples collected in different regions of Brazil. The ability to use any regular real-time thermocycler (without the need of high-resolution melting software) and the simplicity of the test make this protocol easy to implement, allowing its application in influenza surveillance networks worldwide.

## Supplementary Material

Supplemental file 1
