# Supplementary material for: An Inexpensive and Accurate Reverse Transcription-PCR–Melting Temperature Analysis Assay for Real-Time Influenza Virus B Lineage Discrimination
Source: J Clin Microbiol. 2019 Nov 22;57(12):e00602-19. doi: 10.1128/JCM.00602-19 (PMC6879292; doi:10.1128/JCM.00602-19)
Supplement: Supplemental file 1 [file JCM.00602-19-s0001.pdf]

Table S1- GISAID IDs of HA sequences used to verify the lineages characterized by RT-PCR-Tm results.

Victoria Lineage HA Sequences

Isolate ID (GISAID)	Sample ID	Isolate ID (GISAID)	Sample ID
EPI_ISL_11839	B/Brazil/658/2004	EPI_ISL_97383	B/Rio de Janeiro/672/2011
EPI_ISL_75223	B/Bahia/25/2008	EPI_ISL_97384	B/Rio de Janeiro/671/2011
EPI_ISL_75225	B/Bahia/27/2008	EPI_ISL_97385	B/Rio de Janeiro/32/2011
EPI_ISL_75230	B/Minas Gerais/114/2008	EPI_ISL_115044	B/Brazil/2319/2011
EPI_ISL_75231	B/Minas Gerais/117/2008	EPI_ISL_94735	B/Brazil/9442/2011
EPI_ISL_75232	B/Minas Gerais/120/2008	EPI_ISL_94734	B/Brazil/4331/2011
EPI_ISL_75234	B/Minas Gerais/125/2008	EPI_ISL_331415	B/Bahia/1879/2012
EPI_ISL_75235	B/Minas Gerais/129/2008	EPI_ISL_125887	B/Brazil/0944/2012
EPI_ISL_75239	B/Minas Gerais/349/2008	EPI_ISL_146005	B/Rio Grande do Sul/260/2013
EPI_ISL_75249	B/Parana/282/2008	EPI_ISL_145274	B/Santa Catarina/368/2013
EPI_ISL_75252	B/Parana/286/2008	EPI_ISL_145272	B/Santa Catarina/389/2013
EPI_ISL_75253	B/Parana/288/2008	EPI_ISL_145273	B/Santa Catrina/390/2013
EPI_ISL_75254	B/Parana/290/2008	EPI_ISL_331397	B/Parana/1027/2013
EPI_ISL_75255	B/Parana/292/2008	EPI_ISL_145992	B/Brazil/6004/2013
EPI_ISL_75256	B/Parana/294/2008	EPI_ISL_158953	B/Brazil/6828/2013
EPI_ISL_75257	B/Parana/295/2008	EPI_ISL_157841	B/Brazil/3452/2013
EPI_ISL_75260	B/Rio Grande do Sul/157/2008	EPI_ISL_157840	B/Brazil/1036/2013
EPI_ISL_75261	B/Rio Grande do Sul/161/2008	EPI_ISL_147721	B/Brazil/4861/2013
EPI_ISL_75266	B/Rio Grande do Sul/312/2008	EPI_ISL_147720	B/Brazil/3742/2013
EPI_ISL_75268	B/Rio Grande do Sul/316/2008	EPI_ISL_146002	B/Brazil/7795/2013
EPI_ISL_75269	B/Rio Grande do Sul/317/2008	EPI_ISL_164336	B/Brazil/5137/2014
EPI_ISL_75270	B/Rio Grande do Sul/321/2008	EPI_ISL_164325	B/Brazil/2920/2014
EPI_ISL_75272	B/Rio Grande do Sul/327/2008	EPI_ISL_167206	B/Goias/85155/2014
EPI_ISL_75277	B/Rio Grande do Sul/44/2008	EPI_ISL_212809	B/Brazil/0582/2015
EPI_ISL_75278	B/Rio Grande do Sul/47/2008	EPI_ISL_212815	B/Brazil/0596/2015
EPI_ISL_75279	B/Rio Grande do Sul/48/2008	EPI_ISL_214250	B/Brazil/83320/2015
EPI_ISL_75280	B/Rio Grande do Sul/52/2008	EPI_ISL_207364	B/Brazil/80287/2015
EPI_ISL_75282	B/Rio Grande do Sul/55/2008	EPI_ISL_207265	B/Brazil/83320/2015
EPI_ISL_75283	B/Rio Grande do Sul/56/2008	EPI_ISL_207264	B/Brazil/85943/2015
EPI_ISL_75284	B/Rio Grande do Sul/57/2008	EPI_ISL_207256	B/Brazil/87066/2015
EPI_ISL_75296	B/Santa Catarina/150/2008	EPI_ISL_207243	B/Brazil/70230/2015
EPI_ISL_75297	B/Sergipe/258/2008	EPI_ISL_195535	B/Brazil/6297/2015
EPI_ISL_23205	B/Brazil/1143/2008	EPI_ISL_193622	B/Brazil/2786/2015
EPI_ISL_23177	B/Brazil/3636/2008	EPI_ISL_229288	B/Santa Catarina/360/2016
EPI_ISL_32992	B/Brazil/2937/2008	EPI_ISL_229289	B/Parana/121/2016
EPI_ISL_27661	B/Brazil/2937/2008	EPI_ISL_233152	B/Bahia/496/2016
EPI_ISL_25738	B/Brazil/2937/2008	EPI_ISL_233155	B/Parana/1149/2016
EPI_ISL_25736	B/Brazil/4659/2008	EPI_ISL_229285	B/Brazil/02/2016
EPI_ISL_25735	B/Brazil/2937/2008	EPI_ISL_229288	B/Brazil/36/2016
EPI_ISL_97371	B/Sergipe/344/2011	EPI_ISL_229287	B/Brazil/27/2016
EPI_ISL_97372	B/Sergipe/309/2011	EPI_ISL_229289	B/Brazil/121/2016
EPI_ISL_97373	B/Rio de Janeiro/699/2011	EPI_ISL_233152	B/Brazil/0496/2016
EPI_ISL_97374	B/Rio de Janeiro/697/2011	EPI_ISL_233153	B/Brazil/0498/2016
EPI_ISL_97375	B/Rio de Janeiro/696/2011	EPI_ISL_233155	B/Brazil/1149/2016
EPI_ISL_97376	B/Rio de Janeiro/695/2011	EPI_ISL_233154	B/Brazil/0704/2016
EPI_ISL_97377	B/Rio de Janeiro/693/2011	EPI_ISL_238034	B/Distrito Federal/373550/2016
EPI_ISL_97378	B/Rio de Janeiro/681/2011	EPI_ISL_272644	B/Minas Gerais/68/2017
EPI_ISL_97379	B/Rio de Janeiro/677/2011	EPI_ISL_267613	B/Bahia/128/2017
EPI_ISL_97380	B/Rio de Janeiro/676/2011	EPI_ISL_267658	B/Parana/234/2017
EPI_ISL_97381	B/Rio de Janeiro/675/2011	EPI_ISL_296609	B/Santa Catarina/744/2017
EPI_ISL_97382	B/Rio de Janeiro/674/2011	EPI_ISL_292744	B/Parana/967/2017

Yamagata Lineage HA Gene Sequences

Isolate ID (GISAID)	Sample ID	Isolate ID (GISAID)	Sample ID
EPI_ISL_75294	B/Rio de Janeiro/792/2004	EPI_ISL_292745	B/Parana/966/2017
EPI_ISL_75295	B/Rio de Janeiro/809/2004	EPI_ISL_292743	B/Parana/968/2017
EPI_ISL_75224	B/Bahia/26/2008	EPI_ISL_291580	B/Parana/930/2017
EPI_ISL_75226	B/Bahia/301/2008	EPI_ISL_291579	B/Espirito Santo/935/2017
EPI_ISL_75227	B/Bahia/31/2008	EPI_ISL_291578	B/Parana/933/2017
EPI_ISL_75229	B/Minas Gerais/106/2008	EPI_ISL_291577	B/Espirito Santo/934/2017
EPI_ISL_75233	B/Minas Gerais/122/2008	EPI_ISL_291576	B/Rio Grande Do Sul/949/2017
EPI_ISL_75236	B/Minas Gerais/261/2008	EPI_ISL_276549	B/Brazil/520/2017
EPI_ISL_75237	B/Minas Gerais/263/2008	EPI_ISL_276548	B/Brazil/485/2017
EPI_ISL_75238	B/Minas Gerais/264/2008	EPI_ISL_276547	B/Brazil/359/2017
EPI_ISL_75240	B/Minas Gerais/350/2008	EPI_ISL_276546	B/Brazil/346/2017
EPI_ISL_75241	B/Minas Gerais/351/2008	EPI_ISL_276545	B/Brazil/236/2017
EPI_ISL_75242	B/Minas Gerais/352/2008	EPI_ISL_276415	B/Bahia/125/2017
EPI_ISL_75243	B/Minas Gerais/355/2008	EPI_ISL_267660	B/Santa Catarina/207/2017
EPI_ISL_75250	B/Parana/283/2008	EPI_ISL_267659	B/Santa Catarina/145/2017
EPI_ISL_75251	B/Parana/284/2008	EPI_ISL_267612	B/Bahia/127/2017
EPI_ISL_75267	B/Rio Grande do Sul/314/2008	EPI_ISL_267611	B/Bahia/125/2017
EPI_ISL_75271	B/Rio Grande do Sul/324/2008	EPI_ISL_314470	B/Brazil/3250/2017
EPI_ISL_75281	B/Rio Grande do Sul/53/2008	EPI_ISL_313192	B/Brazil/3889/2017
EPI_ISL_97386	B/Bahia/368/2011	EPI_ISL_313109	B/Brazil/6852/2017
EPI_ISL_145276	B/Santa Catarina/374/2013	EPI_ISL_311849	B/Brazil/3018/2017
EPI_ISL_145286	B/Rio de Janeiro/325/2013	EPI_ISL_311556	B/Brazil/1978/2017
EPI_ISL_229286	B/Parana/20/2016	EPI_ISL_305889	B/Brazil/7506/2017
EPI_ISL_300397	B/Santa Catarina/802/2017	EPI_ISL_305188	B/Brazil/8134/2017
EPI_ISL_300396	B/Santa Catarina/447/2017	EPI_ISL_303035	B/Brazil/0459/2017
EPI_ISL_296256	B/Parana/969/2017	EPI_ISL_300974	B/Brazil/9763/2017
EPI_ISL_296254	B/Bahia/975/2017	EPI_ISL_300972	B/Brazil/4261/2017
EPI_ISL_296253	B/Bahia/979/2017	EPI_ISL_300969	B/Brazil/3018/2017
EPI_ISL_292756	B/Parana/715/2017	EPI_ISL_300926	B/Brazil/3889/2017
EPI_ISL_292755	B/Bahia/863/2017	EPI_ISL_287735	B/Brazil/5860/2017
EPI_ISL_292754	B/Bahia/868/2017	EPI_ISL_282030	B/Brazil/9889/2017
EPI_ISL_292753	B/Santa Catarina/914/2017	EPI_ISL_282029	B/Brazil/7440/2017
EPI_ISL_292752	B/Santa Catarina/920/2017	EPI_ISL_282028	B/Brazil/7054/2017
EPI_ISL_292751	B/Santa Catarina/923/2017	EPI_ISL_282027	B/Brazil/3676/2017
EPI_ISL_292747	B/Rio Grande Do Sul/950/2017	EPI_ISL_277648	B/Brazil/9111/2017
EPI_ISL_292746	B/Santa Catarina/959/2017	EPI_ISL_277647	B/Brazil/9090/2017

102 HA sequences from Victoria and 72 from Yamagata samples, totaling 174 sequences used to double check the results obtained with the proposed Sybr-Green based RT-PCR-Tm assay.

FigS1- Amplification curves and melting profiles from 39-replicate obtained with Victoria and Yamagata control strains at 10^{-3} dilution.

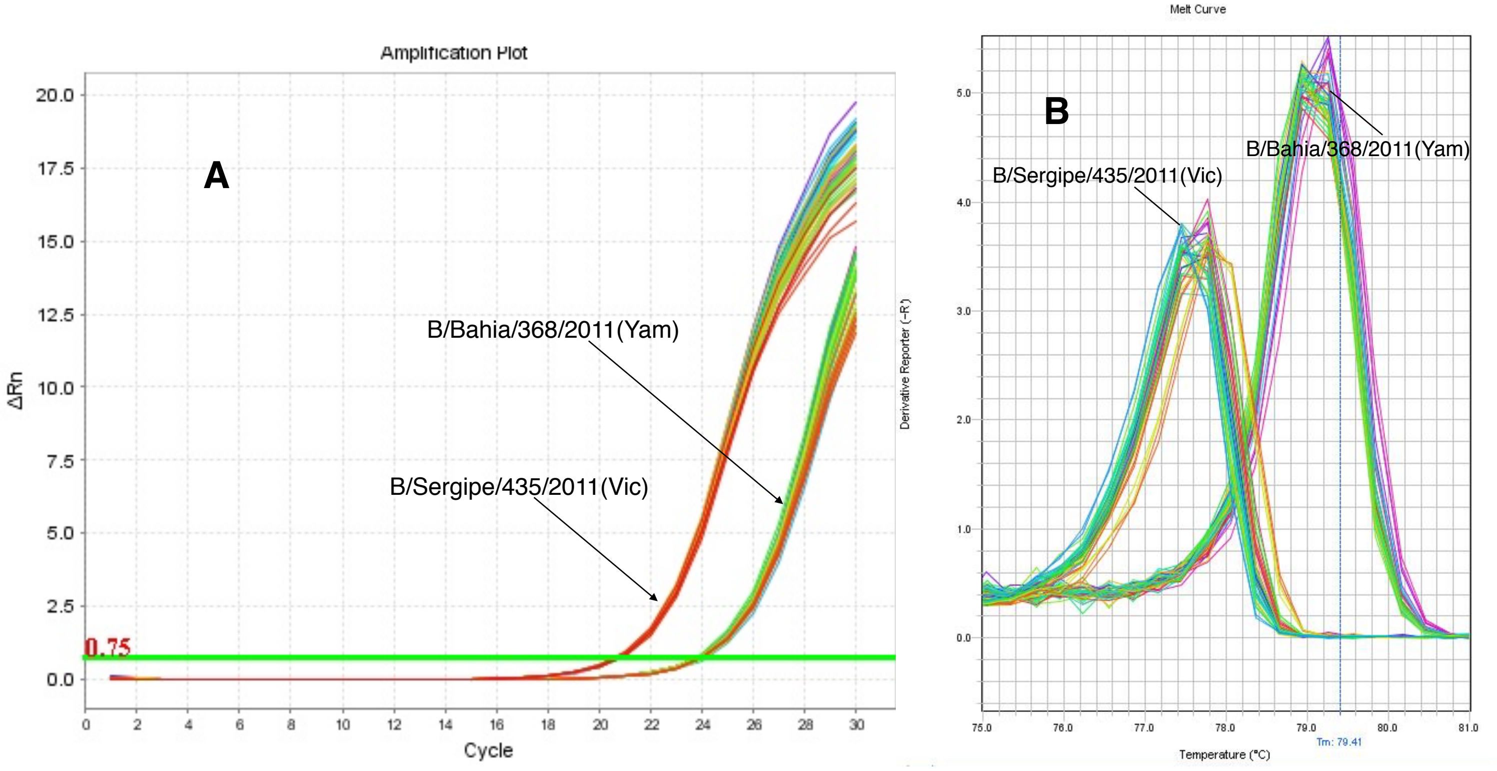

A- The variation between replicates are minimum and the mean Ct in agreement with virus stocks: 20.8 for Yamagata-like sample (33.8 $TCID_{50}$) and 24.0 for Victoria-like (2.94 $TCID_{50}$). **B**- Melting profile for both lineages. The mean Tm are 77.7ºC and 79.2ºC for Victoria and Yamagata controls respectively.

FigS2- Ct value difference against the average of probe-hydrolysis (CDC) and SYBR-Green based RT-PCR-Tm protocol for influenza B detection.

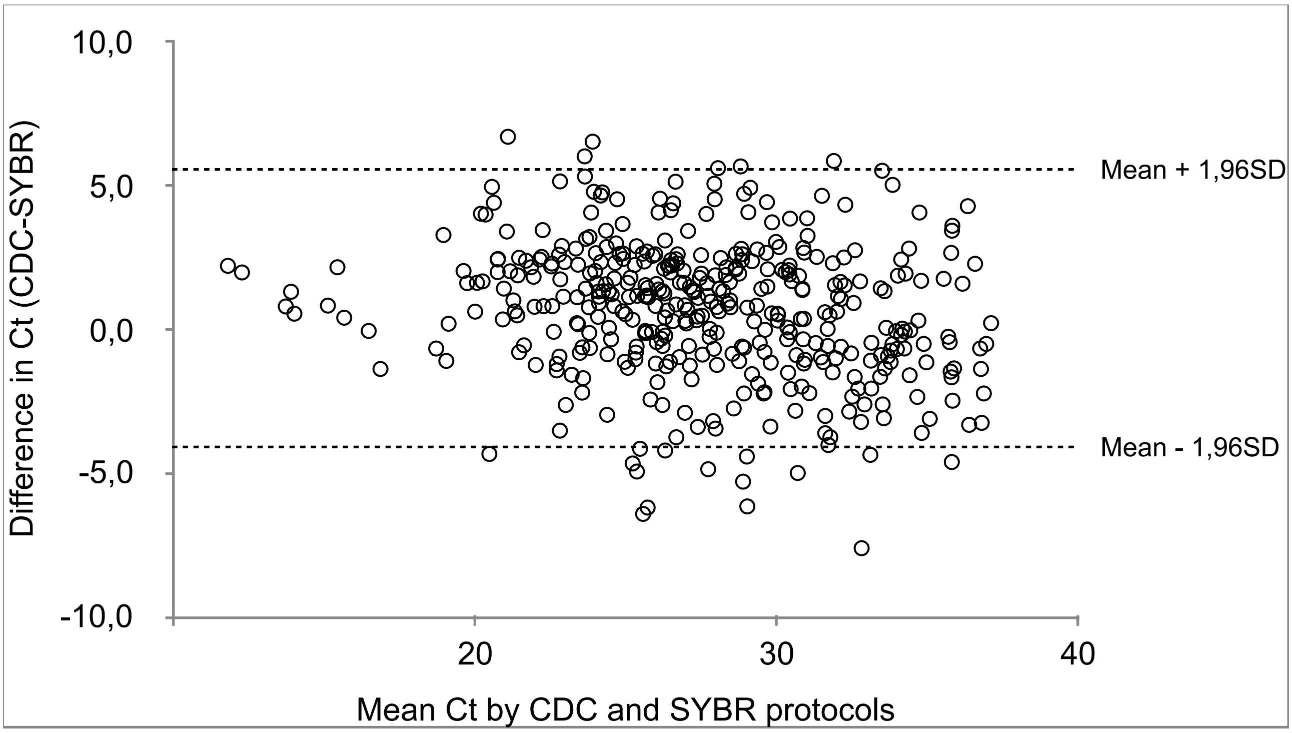

The agreement between the standard probe-hydrolysis (CDC) and the SYBR-Green based protocol for influenza B detection, was assessed by the plotting of Ct difference between both methods (CDC RT-PCR x RT-PCR-Tm), against the Ct mean of each sample (Bland & Altman, 1995). Twenty-three samples (5,6%) out of 410 were outside the 95% limit of agreement (interrupted line), indicating a good correlation between both protocols for influenza B detection.

FigS3- Theoretical denaturing profile of the 131bp HA amplicon of influenza B lineages as function of the temperature.

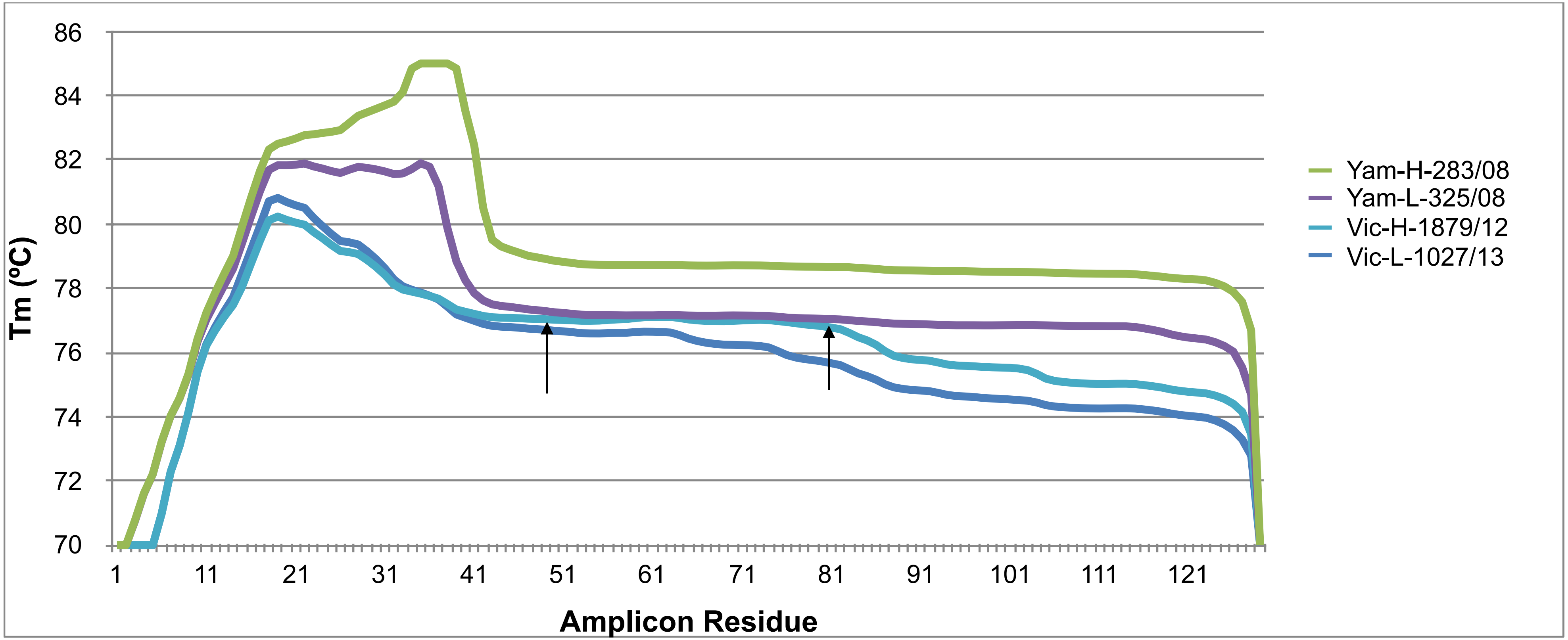

Denaturing profile for Yamagata and Victoria representative sequences with high (Yam-H, Vic-H) and low (Yam-L, Vic-L)Tm profiles were generated using Poland web tool (http://www.biophys.uni-duesseldorf.de/local/POLAND/poland.html). Excepting the region between residues 50 and 80 (between arrows), amplicons from both lineages are clear distinct. The wider variance inside Yamagata lineage is easily observed.

FigS4- Theoretical helicity of Influenza B Victoria and Yamagata lineages amplicons as function of temperature.

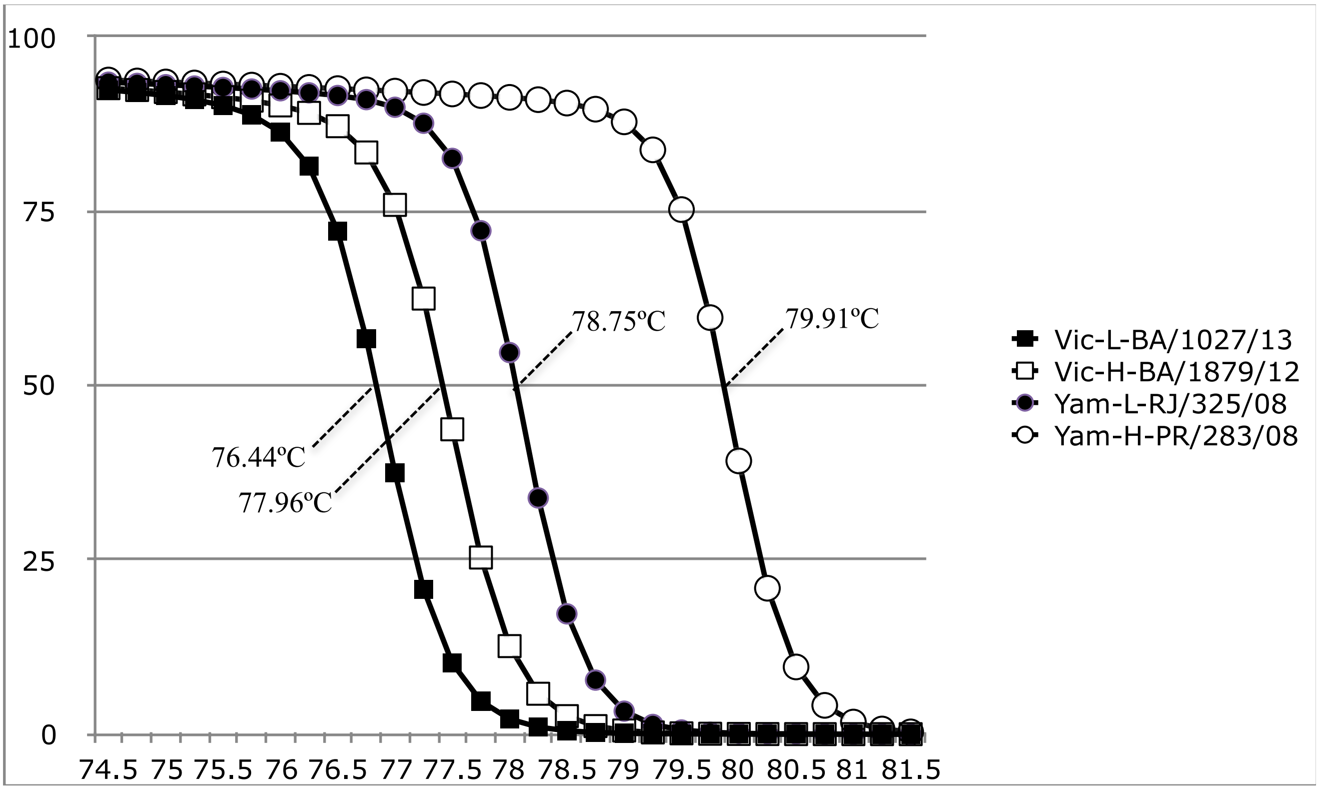

The percentage of double strand amplicons (helicity) as function of the temperature (ºC). Samples B/Bahia/1027/2013 (Vic low Tm), B/Bahia/1879/2012 (Vic high Tm), B/Rio de Janeiro/325/2008 (Yam low Tm) and B/Parana/283/2008 (Yam high Tm) represent extreme Tm observed inside both lineage groups. The variation between samples with low and high Tm was 0.75ºC inside Vic lineage and 1.75ºC for Yam. Tm values are the mean of triplicate assays.
